# Pleuropulmonary Blastoma: A Report of Two Rare Cases

**DOI:** 10.7759/cureus.60021

**Published:** 2024-05-10

**Authors:** Prasath Sathiah, Bheemanathi Hanuman Srinivas

**Affiliations:** 1 Pathology, All India Institute of Medical Sciences (AIIMS) Madurai, Madurai, IND; 2 Pathology, Jawaharlal Institute of Postgraduate Medical Education and Research, Puducherry, IND

**Keywords:** undifferentiated mesenchymal tumor, rhabdomyoblast, pleuropulmonary blastoma, pleural mass, immunohistochemistry

## Abstract

Pleuropulmonary blastoma (PPB) is a rare malignant tumor arising from the lung and pleura. It has three types based on the solid and cystic components. The prognosis of PPB varies depending on the type. Here, we present two female patients who come with complaints of breathlessness. Contrast-enhanced computed tomography (CECT) chest showed a pleural-based mass. Biopsy from the pleural-based mass showed a tumor with features of the malignant mesenchymal tumor. Tumor cells in both cases were positive for vimentin and negative for PanCK. In addition, tumor cells of one case showed positive for BCL2 and α-1 antitrypsin and negative for desmin, CD99, NSE, and p53. Tumor cells of another case are negative for CD99, WT-1, S100, synaptophysin, and chromogranin. In addition, some of the cells have abundant eosinophilic cytoplasm. Desmin shows positive in many cells and highlights rhabdomyoblasts. Morphological and immunohistochemical findings were correlated with the CECT diagnosis of PPB. Both cases were started on neoadjuvant chemotherapy and kept under follow-up. Both the patient's condition improved.

## Introduction

Pleuropulmonary blastoma (PPB) is an uncommon malignant tumor occurring in infancy and childhood and arising from the lung or pleura [1]. Dehner classified PPB as cystic (type I), mixed (type II), or solid (type III) [2]. Currently, the management of PPB depends on the types. Though detecting a DICER 1 mutation is essential in diagnosing PPB, it is not identified in all cases of PPB [3]. Histopathological examination and clinical-radiological correlation help diagnose PPB and exclude the other differential diagnoses. We diagnosed the two cases of PPB during the three-year duration; one was diagnosed as type 2 PPB, and the other was diagnosed as type 3 PPB based on histopathological examination with clinical-radiological correlation.

## Case presentation

Case 1

**Figure 1:**
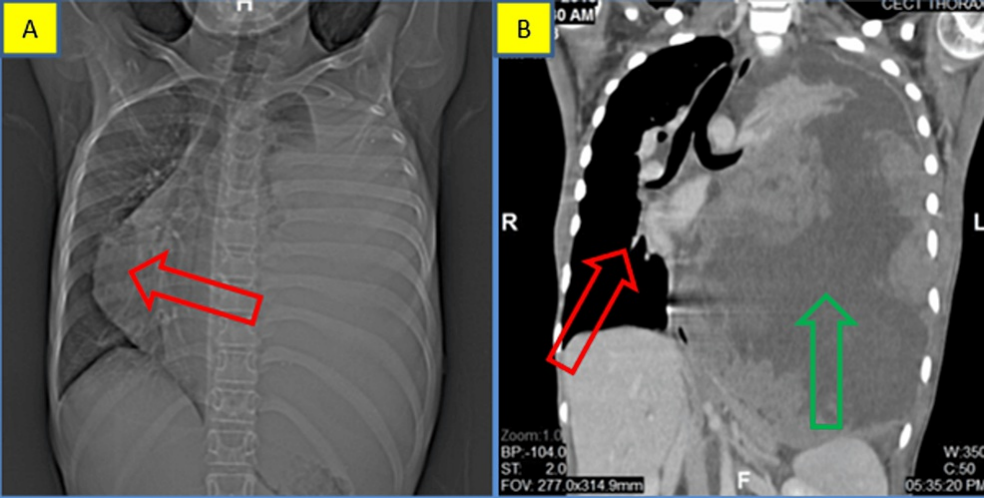
Case 1: radiological features A. Chest radiograph showing an opaque left hemithorax-sparing apex with a mediastinal shift to the right side (red arrow). B. Coronal reformation of computed tomography (CT) showing the mass filling the left lung (blue arrow) and pleural space, displacing the heart to the right side (red arrow).

A 13-year-old female child presented with the chief complaint of breathlessness for one week. A chest radiograph revealed an opaque left hemithorax with a sparing apex and a mediastinal shift to the right side. (Figure 1A). Contrast CT axial sections showing a heterogeneous hypodense mass filling the left lung and pleural space displace the heart to the right side with the collapse of the right lung (Figure 1B).

The mass was heterogeneous and composed of mainly solid areas with some cystic spaces. The CT scan identified no lymph node enlargement. Subsequently, the patient developed a left-sided hemothorax. Intercostal drainage was grossly bloodstained. The microscopic examination of drainage fluid is sterile. The cytological examination of the drained fluid did not reveal any tumor cells. The blood culture did not show any organisms. C-reactive protein value was elevated at 3.6 mg/dl (reference range: less than 0.9 mg/dl) (Table 1).

**Table 1:** Laboratory findings of both cases

Test	Case 1	Case 2
C-reactive protein (reference range: less than 0.9 mg/dl)	3.6 mg/dl	0.8 mg/dl
Blood culture	Sterile	Sterile

**Figure 2:**
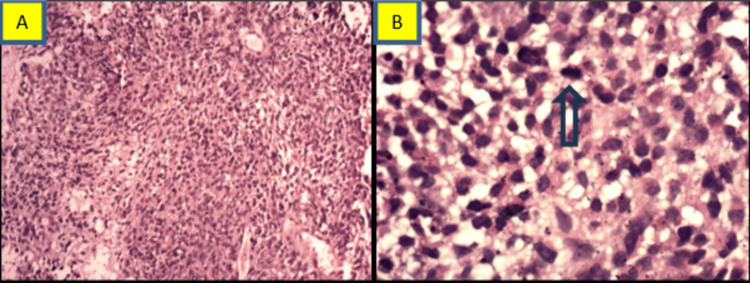
Case 1: light microscopy findings A. Histopathology section showing tumor cells arranged in interlacing fascicles with prominent transversing blood vessels. Tumor cells were oval to spindle-shaped and had a moderate amount of cytoplasm and fine granular chromatin exhibiting mild nuclear pleomorphism (H&E, 100x) (scale bar: 100 µm). B. Arrow in the microsection highlights a mitotic figure (H&E. 400x) (scale bar: 400 µm).

**Figure 3:**
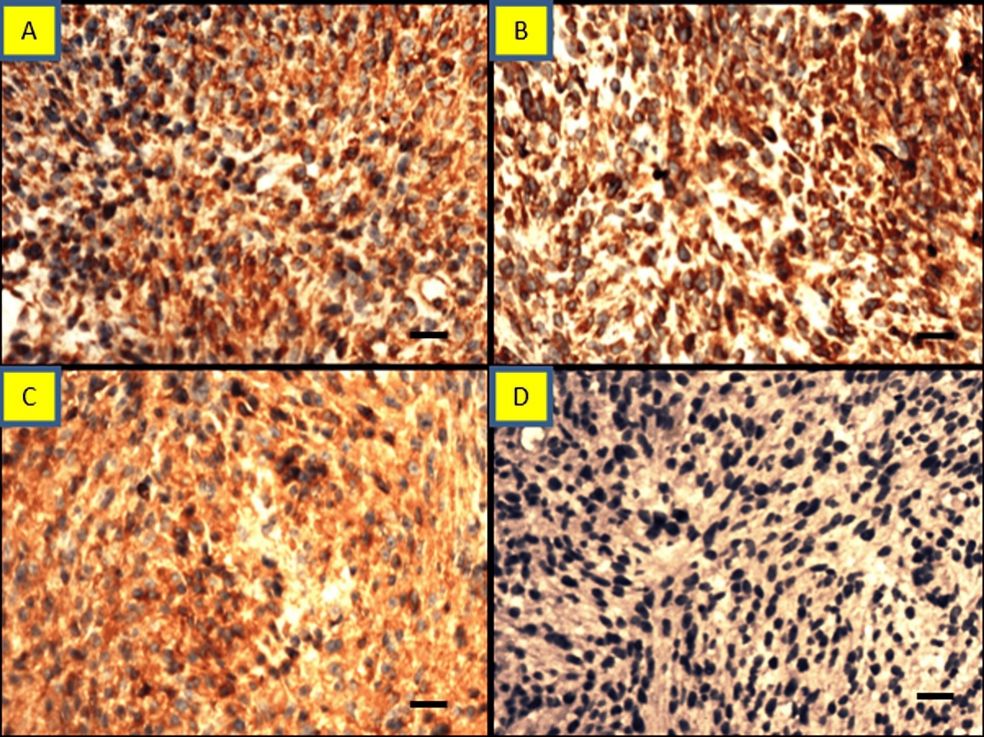
Case 1: immunohistochemistry findings Tumor cells were positive for vimentin (A), BCL2 (B), and alpha-1 antitrypsin(C) and negative for CD117(D) (IHC A-D, 400x) (A-D scale bar: 400 µm).

A trucut biopsy from the left pleural mass showed fragmented tumor tissue arranged in interlacing fascicles with transversing blood vessels. Tumor cells were oval to spindle-shaped, had moderate cytoplasm and fine granular chromatin, and exhibited mild nuclear pleomorphism with mitotic figures (3/10 hpf). In the serial section, we did not identify an entrapped epithelial component, immature cartilage, differentiated rhabdomyomatous component, or necrosis (Figure 2).

Tumor cells were positive for vimentin and negative for PanCK. We did calretinin immunohistochemistry (IHC) because of a pleural-based mass that turned out to be negative. In addition, tumor cells were positive for BCL2 and α-1antitrypsin and negative for desmin, CD99, NSE, and p53. The proliferation index (Ki-67) was 40% (Figure 3).

Morphological features and IHC expression suggested an undifferentiated malignant mesenchymal tumor. Correlating CECT findings (pleural-based mass) with histomorphological and immunohistochemical findings, the final diagnosis was pleuropulmonary blastoma type II (based on the presence of both solid and cystic components in the CECT scan).

Case report 2

**Figure 4:**
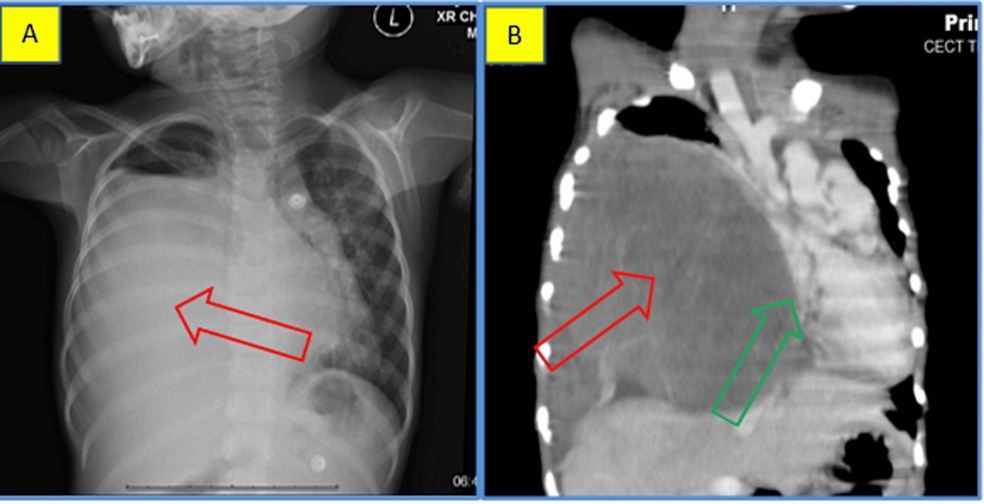
Case 2: radiological features A. Chest radiograph showing an opaque right hemithorax-sparing apex (red arrow) with mediastinal shift to the left side. B. Coronal reformation of CT shows the mass filling of the right lung (red arrow) and pleural space, displacing the heart to the left side (green arrow).

A four-year-old female child presented with a chief complaint of respiratory distress and fever for one week. Chest X-ray showed an opaque, right hemithorax-sparing apex with a mediastinal shift to the left side (Figure 4A). Contrast CT axial sections showed a heterogeneous hypodense mass filling the lung and pleural space, displacing the heart to the left side with the collapse of the lower lobe of the left lung. The mass was composed of predominantly solid areas without any cystic changes (Figure 4B).

The blood culture did not reveal any organisms. The C-reactive protein value was in the normal range, 0.8 mg/dl (reference range: less than 0.9 mg/dl) (Table 1).

**Figure 5:**
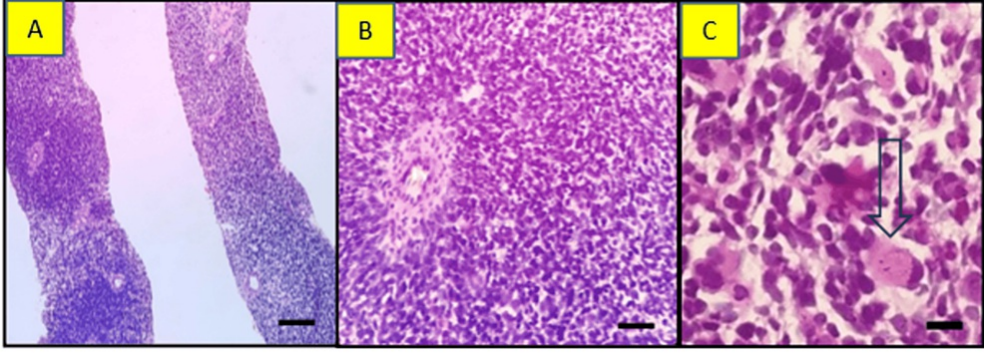
Case 2: light microscopy findings A. Section showing two cores infiltrated by sheets of malignant cells, (H/E, 200x) (scale bar: 200 µm). B. Showing sheets of small round cells with transversing capillaries exhibiting mild anaplasia, (H/E, 400x) (scale bar; 400 µm). C. Showing some cells with abundant eosinophilic cytoplasm with eccentrically placed nuclei (rhabdomyoblasts), (H/E, 400x) (scale bar: 400 µm).

A Trucut biopsy of the mass showed two cores of tissue infiltrated predominantly by sheets of small, round-shaped malignant cells (blastemal elements) with transversing blood vessels. Some tumor cells had oval to spindle cell morphology (malignant mesenchymal components), with scattered tumor cells having abundant eosinophilic cytoplasm with eccentrically placed nuclei reminiscent of rhabdoid cells. Tumor cells were exhibiting mild anaplasia with the presence of mitotic figures (3/10 hpf) (Figure 5).

**Figure 6:**
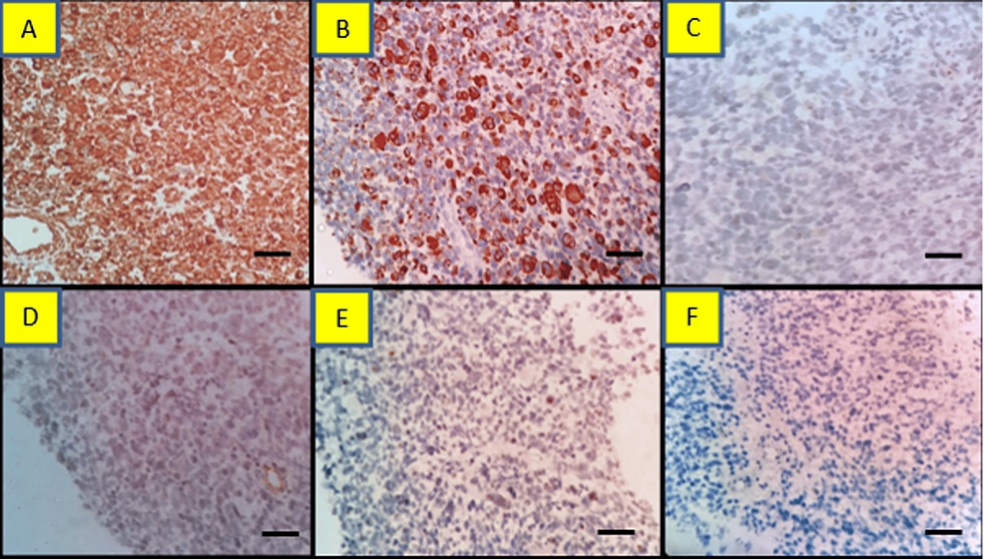
Case 2: immunohistochemistry findings Section showing sheets of malignant cells. A. Positive for vimentin, B. positive for desmin (also highlight the rhabdomyoblasts), C. Negative for Pan CK, D. Focal positive for CD99, E. Negative for WT-1, and F. Negative for synaptophysin (IHC, A-F 400x) (A-F scale bar: 400 µm).

We did not identify necrosis, epithelial elements, or immature cartilage in the serial section. Morphological features suggest the undifferentiated tumor had blastemal and malignant mesenchymal elements. IHC was done, which showed strong vimentin positive in all the tumor cells and negative for PanCK. We used synaptophysin and chromogranin because of the small round cell morphology that returned negative. Desmin was used because scattered cells have abundant eosinophilic cytoplasm that returned positive in many tumor cells and highlighted the rhabdomyoblasts. In addition, tumor cells were negative for CD99, WT-1, and S100 (Figure 6).

The proliferation index was Ki-67 50%. Morphological features and IHC findings suggested a malignant mesenchymal tumor with blastemal and rhabdomyoblastic elements. On correlating with CECT findings, the final diagnosis was pleuropulmonary blastoma type III (based on the presence of predominantly solid components in the CECT scan).

## Discussion

Pleuropulmonary blastoma is an uncommon malignant tumor. It accounts for 0.5% of all malignant tumors in children [4]. The International Pleuropulmonary Blastoma Registry classifies the PPB into three types based on solid and cystic components of tumors [5]. In 2006, based on gross and microscopic examination of specimens, the non-progressive type of PPB was described as PPB Ir [6]. According to the review by Zhang et al., most PPBs reported under six years of age, with no clear-cut gender preferences [7]. Both the present cases are female.

Clinically, the patient may present with nonspecific respiratory symptoms such as chest pain, upper abdominal pain, fever, dyspnea, cough, hemoptysis, anorexia, and neurological symptoms. In addition, PPB type I and type II can present as pneumothorax [8]. Both present cases presented with breathlessness. The first case developed hemothorax on a follow-up scan.

Mutations in DICER 1 occur in 66% of the PPB cases. Patients with this mutation are predisposed to develop malignancy in the kidney, ovaries, peritoneum, brain, and thyroid [3]. Because of a lack of infrastructure and funds, we did not do the DICER 1 mutation. Other system examinations were unremarkable.

A PPB type I tumor is a purely multiloculated cyst lined by cuboidal to the columnar ciliated epithelium. The subepithelium shows undifferentiated round to spindle-shaped cells (cambium layer) with few scattered rhabdomyoblasts. PPB type II and III are differentiated from type I by the presence of a solid component. The solid area comprises blastematous or sarcomatous elements or nodules of cartilage. Even predominantly cystic PPB type II can have a plaque-like or nodular proliferation of cells in the cyst wall. Adequate sampling in PPB type I excludes the possibility of PPB type II [9]. Both the present cases showed solid components. However, the first case is PPB type II because of cystic areas in the CECT scan. However, the presence of infarction, necrosis, or hemorrhage in any of the solid components creates cystic spaces in type III, but these spaces are without epithelium lining.

The presence of blastemal elements excludes the differential diagnosis of rhabdomyosarcoma. WT-1 negative rules out metastatic Wilms tumor. Synovial sarcoma can have a cystic appearance. Immunohistochemistry (positive for epithelial membrane antigen, CD99, and cytokeratin) and molecular study help differentiate synovial sarcoma from PPB. Both the present cases were negative for CD 99 and cytokeratin. A fetal lung interstitial tumor has a monotonous appearance with a structure resembling immature fetal bronchioles, which are absent in the present cases. Solitary fibrous tumors usually have collagenous stroma and staghorn-like vasculature, which are absent in the present cases. Congenital cystic adenomatoid formation IV also has cysts with a thin wall, but it does not have immature nodules or spindle cell collection as seen in PPB type I [10].

Immunohistochemistry has little value in the diagnosis of PPB. Diagnosis is mainly made based on routine histopathological examination [11]. Cytokeratin was positive in the cyst lining epithelium and entrapped epithelium but was negative in both the present cases. Blastemal elements are weakly positive for muscle-specific actin, CD117, alpha-1-antitrypsin, and BCL-2 and negative for WT1. Our first case showed BCL2 and focal alpha-1-antitrypsin positivity, but CD117 was negative. Negative WT1 is helpful to exclude the differential diagnosis of metastatic Wilms tumor. WT-1 is negative in both the present cases. Rhabdomyoblastic elements express desmin, vimentin, and muscle-specific actin; sarcomatous parts express vimentin. Our first case showed positive for vimentin at the sarcomatous component but negative for desmin. The second case showed positive for vimentin and highlighted the rhabdomyoblastic elements with desmin. Cartilaginous parts are positive for vimentin and S100. No cartilaginous part was identified in either of the present cases (S100 negative). There was high proliferative activity in all tumor cell components. Both cases had a high Ki-67 index. P53 expression was associated with the worst prognosis; both the present cases were negative for p53 [12]. It is necessary to correlate the clinical, radiological, histomorphology, and immunohistochemical findings to exclude the possibility of differential diagnosis before labeling the tumor as PPB [10].

Because of aggressive behavior, PPB has the worst prognosis. The International Pleuropulmonary Blastoma Registry reported that overall survival rates for PPB types I, II, and III are 91%, 71%, and 53%, respectively [13]. Patients with mediastinal or extrapulmonary involvement at the time of diagnosis have a worse prognosis than those without such involvement. The common site of metastasis is the brain [13]. So, following up on patients with imaging studies is necessary.

An optimal therapeutic regimen is not available for PPB. PPB type I is treated with surgical excision, and PPB type II and type III tumors are treated with aggressive surgery and chemotherapy. If the cancer is large, neoadjuvant chemotherapy reduces the tumor size [14]. Both cases started on neoadjuvant chemotherapy and were kept under follow-up.

## Conclusions

Various disorders are confused with pleuropulmonary blastoma. It is necessary to diagnose PPB and exclude the possibility of differential diagnosis. It is also essential to type the PPB for treatment and screen the patients for associated malignancies. Radiological features, along with histopathological findings, are essential for diagnosing and typing the pleuropulmonary blastoma.
